# Transcriptome of the egg parasitoid *Fopius arisanus*: an important biocontrol tool for Tephritid fruit fly suppression

**DOI:** 10.1186/s13742-015-0075-4

**Published:** 2015-08-04

**Authors:** Bernarda Calla, Sheina B. Sim, Brian Hall, Theodore DeRego, Guang Hong Liang, Scott M. Geib

**Affiliations:** 1 Tropical Crop and Commodity Protection Research Unit, Daniel K. Inouye United States Pacific Basin Agricultural Research Center, USDA Agricultural Research Services, Hilo, HI USA; 2 Department of Plant and Environmental Protection Sciences, University of Hawaii, Manoa, Honolulu, HI USA; 3 Fujian Agriculture and Forestry University, Forestry College, Fuzhou, China

**Keywords:** Parasitoid wasp, Transcriptome, RNASeq, Hymenoptera, Tephritidae, Biological control, Integrated pest management

## Abstract

**Background:**

The Braconid wasp *Fopius arisanus* (Sonan) has been utilized for biological control of the Mediterranean fruit fly (*Ceratitis capitata*), and the oriental fruit fly (*Bactrocera dorsalis*), both of which are phytophagous fruit fly pests of economic importance in many tropical and subtropical regions of the world. We have sequenced and assembled the transcriptome of this wasp using tissue from four different life stages: larvae, pupae, adult males and adult females, with the aim to contribute foundational resources to aid in the understanding of the biology and behavior of this important parasitoid.

**Findings:**

The transcriptome of the parasitic wasp *Fopius arisanus* was sequenced and reconstructed using a strategy that identified 15,346 high confidence, non-redundant transcripts derived from 8,307 predicted unigenes. In addition, Pfam domain annotations were detected in 78 % of these transcripts. The distribution of transcript length is comparable to that found in other hymenoptera genomes. Through orthology analysis, 7,154 transcripts were identified as having orthologs in at least one of the four other hymenopteran parasitoid species examined. Approximately 4,000 core orthologs were found to be shared between *F. arisanus* and all four of the other parasitoids.

**Conclusions:**

Availability of high quality genomic data is fundamental for the improvement and advancement of research in any biological organism. Parasitic wasps are important in the biological control of agricultural pests. The transcriptome data presented here represent the first large-scale molecular resource for this species, or any closely related Opiine species. The assembly is available in NCBI for use by the scientific community, with supporting data available in GigaDB.

## Data description

### Background

*Fopius arisanus* is an egg-pupal parasitoid of Tephritid fruit flies. It is important as a biological control agent for these invasive and damaging pests stems since it is an egg parasitoid, thus has the ability to infect flies across a broad range of Tephritid speciies during their early developmental stages [1]. In Hawaii, it was estimated that *F. arisanus* constitute up to 95 % of the parasitoid guild, and that levels of parasitism in the oriental fruit fly (*Bactrocera dorsalis*) range between 65 % and 70 %, significantly reducing the infestation of fruits by these flies [2]. However, for some other fly species, such as *Bactrocera cucurbitae* (Melon fly), *F. arisanus* was reported to have low parasitism rates [3, 4]. It is also known that this parasite wasp is able to discriminate between hosts depending on the fruit substrate on which they feed [3]. Foundational genomic and transcriptomic information in this species would help scientists to understand the underlying mechanisms contributing to parasite behavior, describe the physiology and biology of host selection and host–parasitoid interactions, design better biological control strategies, and develop monitoring tools for parasitism rates in the field.

### Samples

Samples were derived from a research colony of *F. arisanus* maintained on *B. dorsalis* at the US Department of Agriculture–Agricultural Research Service (USDA–ARS) Daniel K. Inouye Pacific Basin Agricultural Research Center Insectary in Hilo, Hawaii, USA. Wasp larvae, pupae, and male and female adults were obtained in order to generate samples representative of a broad range of life stages and ages. In brief, a cohort of *B. dorsalis* eggs were exposed to mated *F. arisanus* females for approximately 24 h. Larvae and pupae from the cohort of exposed *B. dorsalis* eggs were dissected in order to target larval and pupal stages of *F. arisanus*. When an *F. arisanus* individual was found, it was carefully removed from the egg, rinsed in sterile water and snap-frozen in liquid nitrogen. Adult males and females were obtained after their emergence from parasitized pupae. For each developmental stage, an effort was made to collect individuals of varying ages within that stage (i.e. corresponding to each developmental instar), so as to encompass as many stage-specific genes as possible. For this purpose, daily collections were made across a developmental stage, total RNA was extracted from each sample, and then RNA samples collected from the same developmental stages were pooled in equimolar concentrations. These samples have been identified as NCBI BioSamples SRS691550, SRS691551, SRS69153, and SRS691554, associated with BioProject PRJNA259570. RNA was extracted from each sample set using the Zymo Quick-RNA MiniPrep Extraction kit (Zymo Research, Irvine, California, USA) following recommended procedures for each tissue. This was then quantified with the Qubit Broad Range RNA assay on a Qubit 2.0 fluorometer (Life Technologies, Carlsbad, California, USA). The size and quality of the total RNA was determined with an RNA 6000 Nano Chip on an Agilent 2100 Bioanalyzer (Agilent Technologies, Santa Clara, California, USA).

### Sequencing

Total RNA was sent to the Beijing Genomics Institute (BGI Americas, University of California, Davis, California, USA) and eukaryotic mRNA libraries were prepared using TruSeq technology (TruSeq RNA Sample Prep Kit v2). The resulting four libraries (larvae, pupae, adult male and adult female) were barcoded and sequenced together on a single lane of the Illumina HiSeq 2000 sequencing system, generating approximately 44.48 Gb of raw data from approximately 211 million 2 × 100 bp-paired reads. These raw reads were filtered by quality and for adapter contamination using an in-house pipeline at BGI, targeting reads containing adapter sequences, those with more than 5 % ambiguous bases, or those with more than 50 % of bases with a Phred quality score below 10. After filtering, data were reduced by approximately 6 % to 42.15 Gb. These filtered data were used for *de novo* assembly, and were also deposited into NCBI under SRA SRX689037, SRX689038, SRX689040, SRX689041, associated with BioProject PRJNA259570.

### Transcriptome assembly

A single representative *de novo* assembly was generated from a concatenation of the four libraries using the Trinity pipeline (r2014_07–17) [5, 6]. In brief, reads were normalized *in silico* to 50x coverage, and then assembled using default Trinity parameters (except for the addition of the ‘--jaccard_clip’ flag to reduce transcript fusions from non-strand-specific data). After assembly, transcript and unigene level expression values were calculated using RSEM [7], and open reading frames (ORFs) were predicted with Transdecoder [6], including those with a detectable Pfam-A domain based on a Hmmer3 search. Next, the raw transcriptome was filtered to discard poorly supported transcripts, and to maintain transcripts with strong evidence of protein coding regions and reasonable support for expression. To do this, we implemented Transvestigator [8], filtering the assembly with parameters set to retain only those transcripts with a transcript per million (TPM) value greater than 0.5, transcript isoforms representing at least 5 % of the abundance of the parent unigene, and transcripts with a predicted ORF. Transvesgitator was also utilized to prepare the data for NCBI Transcriptome Shotgun Assembly (TSA) submission by ensuring that the predicted ORF was on the positive strand. This confirmed a single ORF per transcript, and generated an NCBI .tbl file for submission. In addition to the filters described above, since the larval and pupal samples were derived from the dissection of *B. dorsalis*, any protein sequence with a BLASTp match containing no more than one mismatch at the amino acid level to a *B. dorsalis* protein (acquired from previously published *B. dorsalis* transcriptome and genome datasets, NCBI accessions GAKP00000000.1 and GCF_000789215.1) were flagged and the parent unigene and all transcripts derived from that unigene were discarded. This resulted in the removal of 496 host-derived transcript sequences. Statistics on unfiltered and filtered assemblies are detailed in Table 1.

**Table 1:** Transcriptome assembly and annotation statistics for *F. arisanus*

Number of read pairs used in assembly (SRA accession number)	
Larvae (SRA: SRX689040)	53 174 809
Pupae (SRA: SRX689038)	54 026 754
Adult male (SRA: SRX689037)	53 724 417
Adult female (SRA: SRX689041)	49 823 168
Total	210 749 148
Normalized read pairs (*in silico* normalization)	12 214 054
Unfiltered assembly	
Number of unigenes	57577
N50 unigene length (longest transcript/unigene) (bp)	2162
Sum longest transcript/unigene (Mb)	52.23
Number of transcripts	86118
N50 transcript length (bp)	3174
Sum transcript length (Mb)	117.14
Transcripts per unigene	1.50
GC %	40.45
Filtered *de novo* assembly	
Number of unigenes	8307
N50 unigene length (longest transcript/unigene) (bp)	4751
Sum longest transcript/unigene (Mb)	27.13
Number of transcripts	15346
N50 transcript length (bp)	4570
Sum transcript length (Mb)	50.62
Isoforms per unigene	1.85
GC %	41.37
N50 protein length (amino acids)	282
Number of proteins with complete ORF (%)	11115 (72.4)
Annotation statistics	
Number of proteins with Pfam domains identified	11978
Number of proteins with gene ontology terms	9938
Number of proteins with gene names	14600

### Annotation

Annotation was performed at the peptide level, and these annotations used to generate a transcript name and product, as well as functional annotations. All predicted proteins were subjected to analysis using InterProScan5 to search all available databases, including gene ontology and InterPro term lookup. In addition, proteins were subjected to a BLASTp search against the UniProtKB/SwissProt database (downloaded 10 November 2013). Annotation information was pulled from these results using Annie [8], which assigns gene names and products by cross-referencing SwissProt BLAST hits, and performs database cross-referencing from InterProScan5 results. The resulting annotation file was provided to Transvestigator, as described above, to include functional annotations on the resulting .gff3 and .tbl files (described at [8]).

### Orthology-based comparison of F. arisanus proteins to existing hymenoptera parasitoid genome annotation sets

**Fig. 1:**
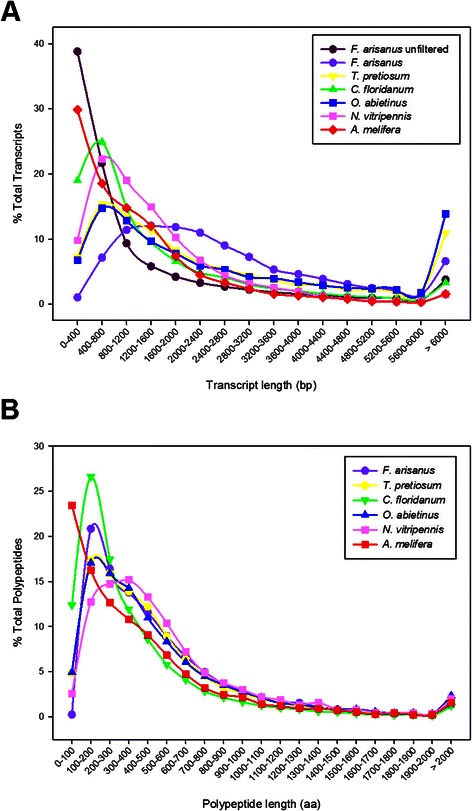
Comparison of *F. arisanus* transcriptome assembly to related hymenopteran parasitoids. Distribution of (**a**) transcript length and (**b**) predicted protein length of the *F. arisanus* transcriptome compared to published transcript and protein sets from related hymenopteran genomes (*Copidosoma floridanum*, *Orussus abietinus* [parasitic wood wasp], *Trichogramma pretiosum*, *Nasonia vitripennis*, and *Apis mellifera*) available on NCBI or the i5k web space (i5k.nal.usda.gov, [9])

**Fig. 2:**
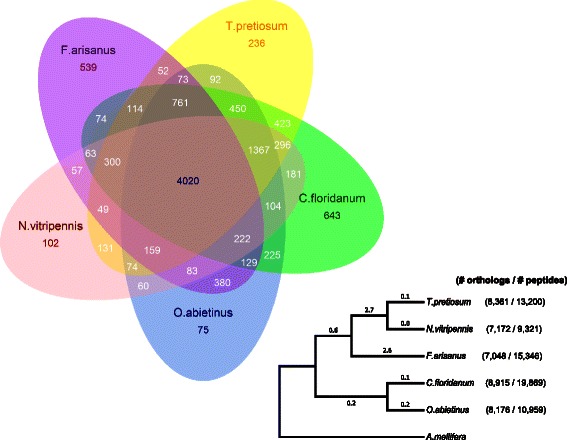
Putative orthologs between parasitoid genomes. Venn diagram showing the number of orthologs shared between five different parasitoid wasp species (*Copidosoma floridanum*, *Orussus abietinus [parasitic wood wasp]*, *Trichogramma pretiosum*, *Nasonia vitripennis*, and *Fopius arisanus*) available on NCBI or the i5k web space (i5k.nal.usda.gov, [9]). Inset tree was constructed utilizing COI (cytochrome c oxidase subunit 1 mitochondrial region) sequences using maximum likelihood and rooted with *A. mellifera* to show relative phylogenetic relatedness of species. Nodes showed >90 % reliability after bootstrapping. Numbers in parentheses after the species name are the number of orthologous proteins (orthologous to at least one of the other species analyzed) and total number of predicted proteins for the respective genome annotations

Transcriptome data were compared with gene sets of four other parasitic wasps: *Copidosoma floridanum* (CFLO draft peptide set, i5k workspace [9])*, Orussus abietinus* (Parasitic Wood Wasp, OABI draft peptide set, i5k workspace), *Trichogramma pretiosum* (TPRE draft peptide set, i5k workspace)*, and Nasonia vitripennis* Jewel Wasp, Nvit_OGSv1, [10]) (Fig. 1)*.* In addition, data from *Apis mellifera* (European Honey Bee, amel_OGSv3.2, [10]) was used to provide comparison with a non-wasp hymenopteran species. Orthologous groups between predicted proteins for these species were identified using OrthoMCL [11, 12] with default parameters. Data were summarized to identify orthologs shared between species (Fig. 2). Peptide sequences for each species, and a putative ortholog list between species, is presented in the GigaDB accession associated with this publication [13].

## Availability of supporting data and materials

The raw datasets supporting the results of this article, including unfiltered assembly results, protein predictions, BLAST results, annotations, and orthology files are available in the *GigaScience* repository [13]. Filtered data used for *de novo* assembly are deposited into NCBI under SRA SRX689037, SRX689038, SRX689040, SRX689041, associated with BioProject PRJNA259570.

## Abbreviations

ORF: Open reading frame
TPM: Transcripts per million
TSA: Transcriptome shotgun assembly
USDA–ARS: United States Department of Agriculture–Agricultural Research Service

## References

[1] Manoukis N, Geib S, Seo D, McKenney M, Vargas R, Jang E. an optimized protocol for rearing *Fopius arisanus*, a parasitoid of tephritid fruit flies. Jove. 2011(53):e2901. doi: 10.3791/2901 10.3791/2901 PMC3196191 21750493

[2] Vargas RI, Leblanc L, Harris EJ, Manoukis NC (2012). Regional suppression of *Bactrocera* fruit flies (Diptera: Tephritidae) in the Pacific through biological control and prospects for future introductions into other areas of the world. Insects.

[3] Bautista RC, Harris EJ, Vargas RI, Jang EB (2004). Parasitization of melon fly (Diptera: Tephritidae) by *Fopius arisanus* and *Psyttalia fletcheri* (Hymenoptera: Braconidae) and the effect of fruit substrates on host preference by parasitoids. Biol Control.

[4] Rousse P, Gourdon F, Quilici S (2006). Host specificity of the egg pupal parasitoid *Fopius arisanus* (Hymenoptera: Braconidae) in La Reunion. Biol Control.

[5] Grabherr MG, Haas BJ, Yassour M, Levin JZ, Thompson DA, Amit I (2011). Full-length transcriptome assembly from RNA-Seq data without a reference genome. Nat Biotech.

[6] Haas BJ, Papanicolaou A, Yassour M, Grabherr M, Blood PD, Bowden J (2013). *De novo* transcript sequence reconstruction from RNA-seq using the Trinity platform for reference generation and analysis. Nat Protocols.

[7] Li B, Dewey C (2011). RSEM. Accurate transcript quantification from RNA-Seq data with or without a reference genome. BMC Bioinformatics.

[8] Hall B, DeRego T, Geib S. Genome Annotation. http://genomeannotation.github.io/.

[9] Poelchau M, Childers C, Moore G, Tsavatapalli V, Evans J, Lee C-Y (2015). The i5k Workspace@NAL—enabling genomic data access, visualization and curation of arthropod genomes. Nucleic Acids Res.

[10] Munoz-Torres MC, Reese JT, Childers CP, Bennett AK, Sundaram JP, Childs KL (2011). Hymenoptera Genome Database: integrated community resources for insect species of the order Hymenoptera. Nucleic Acids Res.

[11] Honeybee Genome Sequencing Consortium (2006). Insights into social insects from the genome of the honeybee *Apis mellifera*. Nature.

[12] Li L, Stoeckert CJ, Roos DS (2003). OrthoMCL: Identification of Ortholog Groups for Eukaryotic Genomes. Genome Res.

[13] Calla B, Sim SB, Hall B, DeRego T, Liang G, Geib SM (2015). Supporting data and materials from “Transcriptome of the egg parasitoid *Fopius arisanus*, an important biocontrol tool for Tephritid fruit fly suppression”. GigaScience Database.

